# Association Between Pancreatic Cysts and Diabetes Mellitus in Von Hippel-Lindau Disease

**DOI:** 10.7759/cureus.54781

**Published:** 2024-02-23

**Authors:** Yasuyuki Onishi, Hironori Shimizu, Sho Koyasu, Daisuke Taura, Ayako Takahashi, Norimitsu Uza, Hiroyoshi Isoda, Yuji Nakamoto

**Affiliations:** 1 Diagnostic Imaging and Nuclear Medicine, Kyoto University, Kyoto, JPN; 2 Von Hippel-Lindau Disease (VHL) Center, Kyoto University Hospital, Kyoto, JPN; 3 Diabetes, Endocrinology and Nutrition, Kyoto University, Kyoto, JPN; 4 Ophthalmology, Kyoto University, Kyoto, JPN; 5 Gastroenterology and Hepatology, Kyoto University, Kyoto, JPN

**Keywords:** serous cysts, simple cysts, von hippel-lindau disease, pancreatic cysts, diabetes mellitus

## Abstract

Introduction: Pancreatic cysts are frequently observed in patients with von Hippel-Lindau disease (VHL), and they are considered clinically not important. This study aimed to evaluate the association between pancreatic cysts and diabetes mellitus (DM) in patients with VHL.

Methods: Among patients who were on a patient list at the VHL Center at Kyoto University Hospital as of December 2022, those who had undergone an upper abdominal magnetic resonance imaging study after 2010 were retrospectively evaluated. The presence or absence of DM and high glycated hemoglobin (HbA1c) levels (>6.0%) were assessed. Patients were divided into two groups: those with DM or high HbA1c levels, and those without DM or high HbA1c levels. The area of the whole pancreas, including the pancreatic cysts and tumors, the area of the pancreatic cysts, and the percentage of pancreatic cysts, calculated by dividing the area of pancreatic cysts by the area of the whole pancreas, were measured on T2-weighted magnetic resonance images and compared between the two groups.

Results: Thirty-six patients with VHL, comprising 22 men and 14 women, with a mean age of 36.4 years (range, 11-79 years), were identified. Seven patients had DM, and two additional patients had high HbA1c levels. The area of the pancreatic cysts (p = 0.0013) was significantly larger and the percentage of the pancreatic cysts (p = 0.0016) was significantly higher in patients with DM or high HbA1c levels (n = 9) than in patients without DM or high HbA1c levels (n = 27); however, the difference in the area of the whole pancreas was not significant (p = 0.068).

Conclusion: Our findings suggest that patients with VHL who have a large area covered by pancreatic cysts are more likely to have DM than those without.

## Introduction

Von Hippel-Lindau disease (VHL) is an autosomal dominant disorder that affects one in 36,000 live births [1]. Various types of tumors, such as hemangioblastomas of the central nervous system (CNS) and retina, clear cell renal cell carcinomas, pheochromocytomas, paragangliomas, and pancreatic neuroendocrine tumors, can develop in patients with VHL [1]. As these tumors are clinically important, their surveillance is recommended [2]. Pancreatic cysts are frequently observed in patients with VHL and can be classified into simple cysts (also known as true cysts and serous cysts) or serous cystadenomas [3-5]. Pancreatic cysts do not result in clinical complications and are generally considered clinically insignificant [3,5,6]. However, few studies have reported that multiple pancreatic cysts may cause diabetes mellitus (DM) [7-9]. The objective of this study was to evaluate the association between pancreatic cysts and DM in patients with VHL.

## Materials and methods

Patients

This retrospective study was approved by our Institutional Review Board (R4100), and the requirement for obtaining informed consent was waived due to the retrospective nature of the study. Our institution has a clinical center dedicated to VHL care recognized by the VHL Alliance, comprising multiple departments, such as ophthalmology, neurosurgery, urology, gastroenterology, pancreatic surgery, endocrinology, and diagnostic radiology. The VHL center maintains a patient list to provide genetic care to patients with VHL, including healthcare and genetic counseling for the whole family. Among the patients with VHL on the list as of December 2022, those who had undergone an upper abdominal magnetic resonance imaging (MRI) study after 2010 were selected for inclusion in this study. Patients with a history of pancreatic resection were excluded from the study unless an upper abdominal MRI meeting the following two conditions was available: (1) MRI was performed after 2010 and (2) MRI was conducted before pancreatic resection.

Diagnostic criteria for VHL

Patients with a family history of VHL who had ≥1 VHL-related tumor, such as CNS/retinal hemangioblastomas, pheochromocytomas, renal cell carcinomas, or pancreatic neuroendocrine tumors, were diagnosed with VHL. Patients who did not have a family history of VHL were diagnosed with VHL if they had ≥ two CNS/retinal hemangioblastomas or one CNS/retinal hemangioblastoma and a VHL-related visceral tumor [10].

Diagnostic criteria for DM

Patients with a glycated hemoglobin (HbA1c) level of ≥6.5% or those who met the 1999 World Health Organization criteria for DM (i.e., a fasting plasma glucose level of ≥126 mg/dL and/or a two-hour glucose level of ≥200 mg/dL in the 75-gram oral glucose tolerance test) were diagnosed with DM. High HbA1c levels were defined as HbA1c levels >6.0% but <6.5%. The presence or absence of DM or high HbA1c levels at the time of the most recent upper abdominal MRI study was assessed. In patients with a history of pancreatic resection, the presence or absence of DM or high HbA1c levels at the time of the most recent upper abdominal MRI before pancreatic resection was assessed.

MRI technique

MRI examinations were performed using a 1.5- or 3-T system. Although various protocols and imaging parameters were used during the MRI studies, the breath-hold axial heavily T2-weighted (T2W) imaging with a half-Fourier single-shot turbo spin-echo sequence or fat-saturated axial T2W imaging with turbo spin-echo sequence were undertaken for the patients included in this study. In addition, the breath-hold axial 3D T1-weighted (T1W) imaging with a gradient echo sequence was undertaken.

Image evaluation

**Figure 1:**
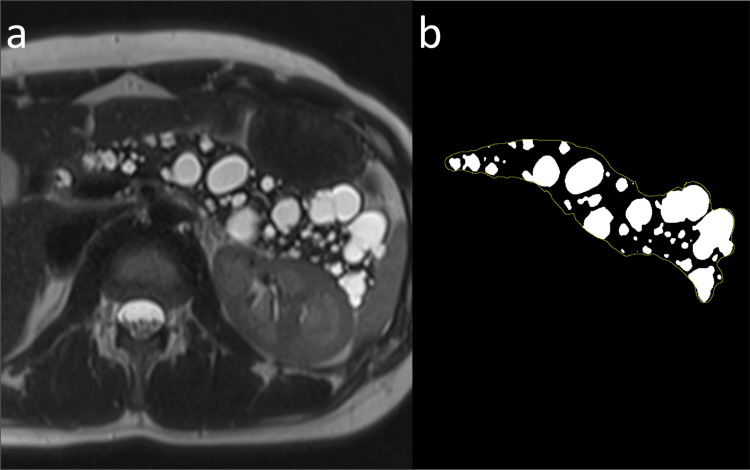
Measurement of pancreatic cysts in a 25-year-old woman without diabetes mellitus or high glycated hemoglobin level (a) An axial heavily T2-weighted magnetic resonance image that maximized the total area of the pancreatic body and tail is selected for each patient. (b) The image is changed to a black and white display according to a cut-off value. The cut-off value is adjusted such that the pancreatic cysts appear white and the regions without cysts appear black. The percentage of pancreatic cysts is 48.1%.

The MRI images from the most recent upper abdominal MRI study were evaluated by a board-certified radiologist with 12 years of experience in abdominal imaging (Y.O.). In the case of patients who had undergone surgical resection of the pancreas, images from the most recent upper abdominal MRI study obtained before pancreatic resection were used for evaluation. Axial heavily T2W images of the upper abdomen were reviewed, and the image that maximized the total area of the pancreatic body and tail, including the pancreatic cysts and tumors, was selected. The area of the whole pancreas, including the cysts and tumors, was measured on the image by manually tracing the contour of the pancreas using ImageJ software (version 1.53 K, NIH, Bethesda, MD). Information beyond the pancreas was cleared from the axial image, and the image was converted into a black-and-white display according to a cut-off value. Pixels with MRI signal values above the cut-off value were displayed as white, whereas those with MRI signal values below the cut-off value were displayed as black. Subsequently, the cut-off value was adjusted such that pancreatic cysts appeared white and the regions without cysts appeared black. The area of the white regions was calculated to determine the area of the pancreatic cysts (Figure 1). The percentage of the pancreatic cysts was defined as the area of the pancreatic cysts divided by the area of the whole pancreas. Fat-saturated axial T2W images were used for evaluation if axial heavily T2W images were unavailable. The contour of the pancreas enclosed in the T2W image was verified using axial T1W images. In addition, the presence of dilatation of the main pancreatic duct, defined as a diameter of >4 mm, was evaluated.

Statistical analysis

Statistical analyses were performed using JMP software (version 16.2.0; SAS Institute, Cary, NC). Statistical significance was set at p < 0.05. Patients were divided into two groups: those with DM or high HbA1c levels and those without DM or high HbA1c levels. Differences in age, area of the whole pancreas, area of the pancreatic cysts, and percentage of area occupied by pancreatic cysts were evaluated using the Mann-Whitney U test between the two groups. The presence or absence of dilatation of the main pancreatic duct was compared between the two groups using Fisher’s exact test.

## Results

Seventy-four patients with VHL were identified from the patient list of the VHL center. Among these 74 patients, 36 who did not undergo an MRI study of the upper abdomen after 2010 were excluded. Of the remaining 38 patients, four had undergone surgical resection of the pancreas for neuroendocrine tumors (n = 3) and metastatic renal cell carcinoma (n = 1). In two of them, upper abdominal MRIs obtained before pancreatic resection and after 2010 were not available, and they were excluded. Thus, 36 patients (22 men and 14 women) were included in this study. The mean age of the patients at the time of undergoing MRI study was 36.4 years (range, 11-79 years). All patients had a history of VHL-associated tumors (Table 1).

**Table 1:** History of VHL-associated tumors in the 36 patients VHL: von Hippel-Lindau disease.

	Number of patients
Central nervous system hemangioblastoma	32
Retinal hemangioblastoma	23
Renal cell carcinoma	15
Pancreatic neuroendocrine tumor	14
Pheochromocytoma	4

Twenty-one patients had a family history of VHL. The body mass index (BMI) within six months of the most recent abdominal MRI study was available for 19 patients. The mean BMI was 22.1 kg/m2 (range, 13.7-28.7 kg/m2).

Seven patients were diagnosed with DM at the time of the most recent abdominal MRI. Among these seven patients, four had insulin-dependent DM and three had insulin-independent DM. In addition to these patients, two patients had high HbA1c levels (6.3 and 6.4 mg/dl). The remaining 27 patients did not have DM or high HbA1c levels.

**Figure 2:**
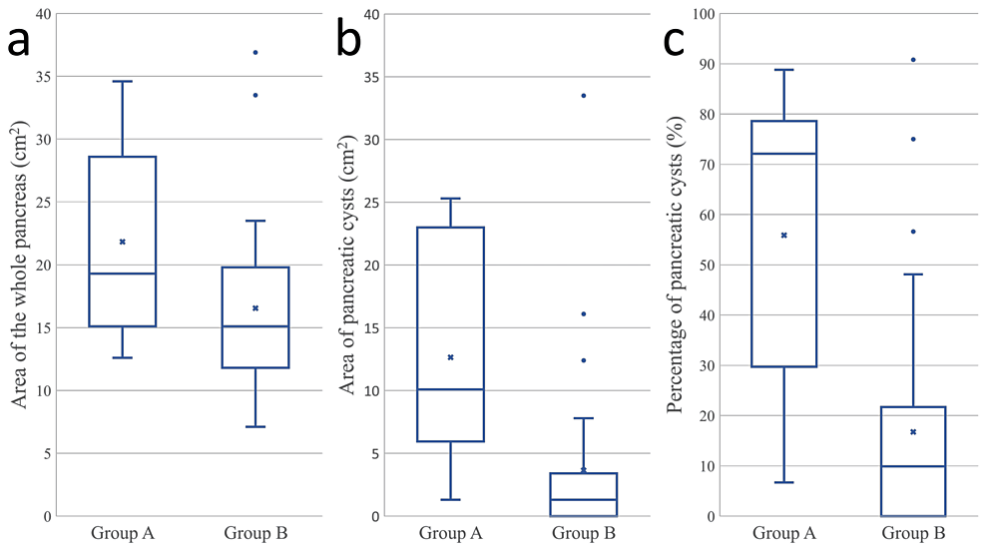
Box plots comparing the measurements of the pancreas between the two groups (a) The area of the pancreas. (b) The area of the pancreatic cysts. (c) The percentage of pancreatic cysts. Group A: patients with DM or high HbA1c levels. Group B: patients without DM or high HbA1c levels. DM, diabetes mellitus; HbA1c, glycated hemoglobin.

**Figure 3:**
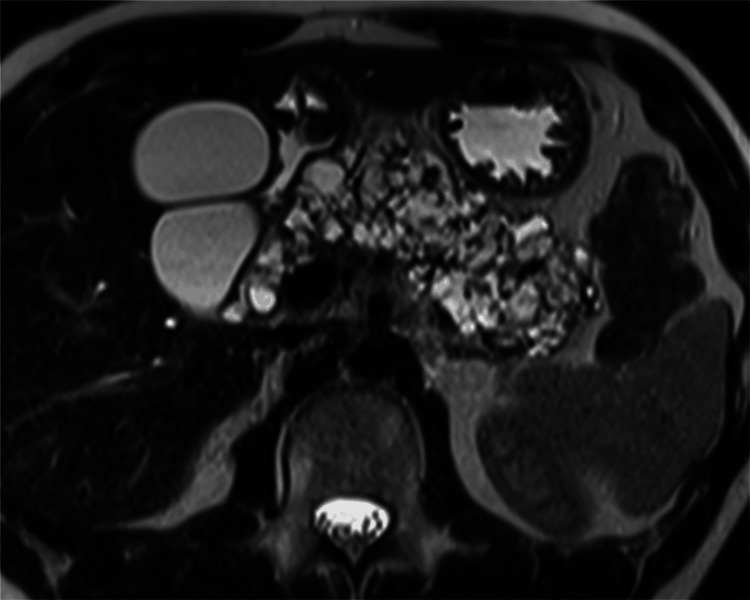
An axial heavily T2-weighted magnetic resonance image of a 36-year-old man with insulin-dependent diabetes mellitus (Patient 4 in Table 3) The percentage of pancreatic cysts is 72.1%.

Table 2 presents the results of the imaging evaluation of the pancreas. The differences in the imaging findings between the two groups are illustrated in Figure 2. Patients with DM or high HbA1c levels (n = 9) had a significantly larger area of pancreatic cysts (p = 0.0013) and a significantly higher percentage of pancreatic cysts (p = 0.0016) than patients without DM or high HbA1c levels (n = 27). Table 3 summarizes the findings of all patients with DM or high HbA1c levels (Figure 3). The mean age of these patients was 39.4 years (range, 23-58 years). Seven patients were diagnosed with DM at a mean age of 30.7 years (range, 9-47 years).

**Table 2:** Measurement of the pancreas Differences in age, area of the whole pancreas, area of the pancreatic cysts, and percentage of area occupied by pancreatic cysts were evaluated using the Mann-Whitney U test between the two groups. The presence or absence of dilatation of the main pancreatic duct was compared between the two groups using Fisher’s exact test. DM: diabetes mellitus; HbA1c: glycated hemoglobin.

	All patients (n = 36)	Patients with DM or high HbA1c levels (n = 9)	Patients without DM or high HbA1c levels (n = 27)	p
Age (years) (mean)	36.4	39.4	35.4	0.3903
Area of the whole pancreas (cm^2^) (mean, range)	17.9 (7.1–36.9)	21.8 (12.6–34.6)	16.5 (7.1–36.9)	0.0677
Area of pancreatic cysts (cm^2^) (mean, range)	5.9 (0–33.5)	12.6 (1.3–25.3)	3.6 (0–33.5)	0.0014
Percentage of pancreatic cysts (%) (mean, range)	26.5 (0-90.8)	55.9 (6.7–88.8)	16.7 (0–90.8)	0.0016
Dilatation of the main pancreatic duct	2	0	2	1

**Table 3:** Patients with diabetes mellitus or high HbA1c (n = 9) HbA1c: glycated hemoglobin; CNSHB: central nervous system hemangioblastoma; DM: diabetes mellitus; MPD: main pancreatic duct; NET: neuroendocrine tumor; Pheo: pheochromocytoma; RCC: renal cell carcinoma; RHB: retinal hemangioblastoma.

Cases	Age (years)/ Sex	DM	Insulin-dependent	Age at DM diagnosis (years)	Body mass index (kg/m^2^)	Area of the whole pancreas (cm^2^)	Area of pancreatic cysts (cm^2^)	Percentage of pancreatic cysts (%)	Dilatation of the MPD	Pancreatic NETs	Other tumors
1	23/M	Yes	Yes	9	22.2	28.5	25.3	88.8	No	Yes	CNSHB, RCC, RHB
2	23/F	Yes	No	22	30.6	16.3	7.5	46.0	No	Yes	CNSHB, RCC, RHB
3	30/M	Yes	Yes	26	26.6	34.6	25.3	73.1	No	No	CNSHB, RHB
4	36/M	Yes	Yes	23	N.A.	28.7	20.7	72.1	No	No	CNSHB, RHB
5	40/M	No	No	N.A.	23.7	12.6	10.6	84.1	No	Yes	CNSHB, RCC, RHB
6	41/M	Yes	No	40	25.5	25.2	8.6	34.1	No	No	CNSHB, RCC
7	50/M	Yes	Yes	47	22.4	19.3	1.3	6.7	No	Yes	CNSHB, RHB
8	54/M	No	No	N.A.	28.7	17.4	4.4	25.3	No	No	CNSHB, Pheo, RCC
9	58/F	Yes	No	48	N.A.	13.9	10.1	72.7	No	Yes	CNSHB

**Figure 4:**
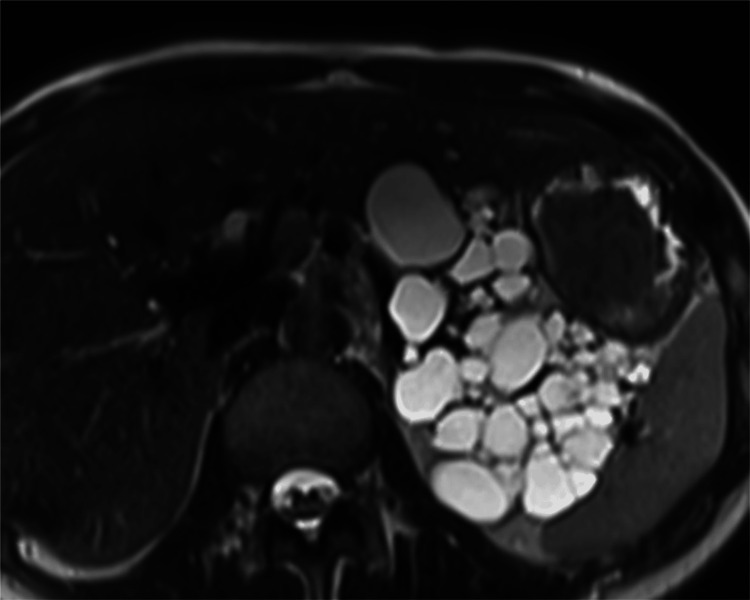
An axial heavily T2-weighted magnetic resonance image of a 25-year-old woman without diabetes mellitus or a high HbA1c level This patient is different from the patient in Figure 1. The percentage of pancreatic cysts is 90.8%.

Among the nine patients with DM or high HbA1c levels, the percentage of pancreatic cysts was ≥50% in five patients (55.6%). Among the 27 patients without DM or high HbA1c levels, the percentage of pancreatic cysts was ≥50% in three patients (11.1%) (Figure 4). This frequency (11.1%) was much lower than that (55.6%) in patients with DM or high HbA1c levels.

## Discussion

This study examined the association between the area occupied by the pancreatic cysts and DM in 36 patients with VHL. DM was observed in seven of the 36 patients (19.4%). Considering the relatively young age of the study population (mean age, 36.4 years), the frequency was much higher than that in the general Japanese population (7.9%) [11]. The area of the pancreatic cysts was larger and the percentage of the pancreatic cysts was higher in patients with DM or high HbA1c levels than in patients without DM or high HbA1c levels in this study. Thus, it was speculated that the presence of pancreatic cysts may affect the endocrine function of the pancreas and that patients with VHL who have a large area occupied by pancreatic cysts are more likely to have DM than patients without pancreatic cysts. This speculation differs from the findings of several previous studies that pancreatic cysts rarely cause symptoms [3,5,6,12-14]. A few studies have reported that multiple pancreatic cysts may cause DM in patients with VHL [7-9]. A previous study reported that DM is the most common clinical sign in patients with extensive serous microcystic adenomas [7]. Another study reported that DM was observed in three out of 11 patients (27%) and recommended regular screening and follow-up for DM [15]. Since the association between the pancreatic cysts and DM in patients with VHL observed in this study is in contrast with the results presented in previous studies, this association should be considered with caution. This discrepancy could be due to the small sample size and lack of detailed information regarding glucose metabolism at the time of DM diagnosis in the present study. Thus, further studies with larger sample sizes and more detailed information regarding glucose metabolism are necessary to evaluate the association between pancreatic cysts and DM in patients with VHL. If such an association is identified, routine screening for DM in patients with VHL who have many pancreatic cysts can lead to an early diagnosis of DM.

This study has several limitations. First, the study population was small owing to the rarity of VHL. Second, the MRI scanner and sequences used in this study were not consistent. Third, the measurement of areas occupied by pancreatic cysts may not be accurate. For example, pancreatic cysts may show low signal intensity depending on the nature of the fluid, whereas cystic pancreatic neuroendocrine tumors may appear as pancreatic cysts. Fourth, the study population was limited to those who underwent upper abdominal MRI studies, which may have led to selection bias. Fifth, most patients were diagnosed with DM at other hospitals, and there was no information regarding type 1 DM.

## Conclusions

Although the sample size was not large owing to the rarity of VHL, the area covered by the pancreatic cysts was larger and the percentage of the pancreatic cysts was higher in patients with VHL who had DM or high HbA1c levels compared with those in patients without DM or high HbA1c levels. The findings of the present study suggest that patients with VHL who have a large area occupied by pancreatic cysts are more likely to have DM than those without.
